# X-ray-Fluorescence-Based Screening Method for Uranium in Contaminated Brackish Water Using Graphene Oxide Nanosheets

**DOI:** 10.3390/membranes13030299

**Published:** 2023-03-03

**Authors:** Hiroshi Yoshii, Kodai Takamura, Tetsuaki Uwatoko, Yasuhiro Sakai

**Affiliations:** 1 National Institute of Radiological Science, National Institutes for Quantum Science and Technology, 4-9-1 Anagawa, Inage-ku, Chiba 263-8555, Japan; 2 Department of Physics, Faculty of Science, Toho University, 2-2-1 Miyama, Funabashi 274-8510, Japan

**Keywords:** uranium contamination, brackish water, X-ray fluorescence, salinity

## Abstract

In the event of uranium release into the environment due to an accident, confirming the presence of uranium contamination is difficult because uranium is a naturally occurring element. In this study, we developed a method based on X-ray fluorescence (XRF) for the facile screening of uranium in brackish water samples in the event of an accident in a coastal area. Graphene oxide nanosheets were added to uncontaminated brackish water sampled from different sites to adsorb the uranium present in the samples, if any. The graphene oxide nanosheets were then collected using a membrane filter and analyzed using XRF. The results revealed that the signal intensity of the U Lα peak was proportional to the salinity. Hence, uranium contamination could be confirmed when the intensity of the U Lα peak was significantly greater than that derived from the background uranium content, as estimated from the salinity value. Thus, in the event of an accident, the salinity of the collected brackish water should be measured, and XRF analysis should be performed using our developed method. This method is useful for screening brackish water for uranium contamination.

## 1. Introduction

Uranium, which is the main component of nuclear fuel and has the largest weight ratio of spent nuclear fuel, exhibits various toxicity levels when it enters the human body [1,2,3]. In the event of a large-scale accident at a nuclear fuel handling facility such as a nuclear power plant, uranium may be released into the environment. In fact, it has been confirmed that following the Fukushima Daiichi Nuclear Power Station accident caused by the Great East Japan Earthquake in 2011, uranium was scattered not only throughout the premises, but also over a wide area [4,5]. In addition to accidents during the transportation of nuclear fuel and spent nuclear fuel [6], the use of depleted uranium ammunition may also result in the release of uranium into the environment [7,8]. In such a case, for health and safety reasons, it is paramount that residents understand and swiftly react to the situation. If spent nuclear fuel is released into the environment, its presence can be determined by measuring the γ-rays emitted by fission products, but in the case of pre-service nuclear fuel or depleted uranium munitions, the detection of uranium itself is necessary. However, because uranium is a naturally occurring element, any analysis of environmental samples is skewed by the presence of natural uranium. Because the concentration of natural uranium varies significantly between environmental samples, it is difficult to confirm the presence of contamination after an accident. Thus, post-accident environmental samples that can provide an estimation of naturally occurring uranium through the use of adequate indices are promising for confirming the presence or absence of uranium contamination. In this regard, brackish water close to the estuary of a river is a potential candidate because it can be used to determine the concentration of naturally occurring uranium; this process is described in detail below. Additionally, because nuclear facilities are often located in coastal areas, brackish water analysis is a promising method for accident-derived uranium determination.

Although the uranium concentration in seawater is generally stable worldwide at ~3.2 ng mL^−1^ [9], in Japanese river water, it falls within the range of ~0.1–600 pg mL^−1^ [10,11,12], and changes with the tide in brackish water. Moreover, although the behavior of uranium close to the estuary is complex, the uranium concentration generally correlates with the salinity level [13,14,15]. Therefore, we expected the determination of the salinity of brackish water to provide an indication of the original uranium concentration prior to an accident. Even though highly saline samples, such as seawater and brackish water, cannot be subjected to analysis by inductively coupled plasma mass spectrometry (ICP-MS) [16], X-ray fluorescence (XRF) analysis can be employed. Indeed, we recently proposed a new method for the XRF-based analysis of trace uranium in sodium perchlorate aqueous solutions with added uranium [17]; specifically, we proposed that the uranium present in the sample solution adsorbs onto the surface of graphene oxide (GO) nanosheets in the presence of sodium perchlorate, thereby promoting GO aggregation [18,19,20,21,22] and preventing pore blockage during subsequent sample collection using a membrane filter. Our previous study indicated that the optimal amount of sodium perchlorate resulted in a salinity reading of 3–4% [17]. It should be noted that salinity is generally expressed as a dimensionless quantity in permillage; however, the readings of the salinity meter employed in this study are displayed as “% (*w*/*w*)”. As the salinity of seawater is 3.1–3.8%, the analytical result is not affected, and indeed, the aggregation of GO is promoted. As a result, our recently proposed method can be applied to seawater, brackish water, and river water to which sodium perchlorate has been added.

Thus, in the present study, samples of seawater, brackish water, and river water with various salinities were collected and analyzed via XRF, following the GO-adsorption method described above. Subsequently, the correlation between salinity and the signal intensity of the U Lα peak was investigated, and a method was developed to confirm whether the uranium concentration in brackish water increased. In our previous study [17,22], small sample volumes were employed; by contrast, relatively large volumes of brackish water were collected for the current study. Furthermore, we investigated the optimal quantity of dispersed GO required for uranium adsorption. To examine the inhibition of uranium adsorption onto GO by the matrix components in the seawater samples, the standard addition and calibration curve methods were compared.

## 2. Materials and Methods

### 2.1. Reagents and Materials

All chemicals used for XRF analysis were of analytical grade. High-purity water was prepared using a Milli-Q^®^ Integral 5 ultrapure water purification system (Merck & Co., Inc., Rahway, NJ, USA). Sodium perchlorate monohydrate (FUJIFILM Wako Pure Chemical Corporation, Osaka, Japan) was used to adjust the sodium ion concentration. A GO nanosheet (size ~600 nm, 4 mg mL^−1^, dispersed in H_2_O, Merck & Co., Inc., Rahway, NJ, USA) was used to adsorb uranium. Similarly to our previous studies [17,22,23,24], the multielement standard XSTC-1407 (Spex CertiPrep Inc., Metuchen, NJ, USA), containing 10 μg mL^−1^ of each element (Co, Cs, Cu, Th, and U), was employed to adjust the uranium concentration in the standard sample. To obtain the fitting parameter for the Br Kβ peak, a 0.05 mol L^−1^ bromine solution (FUJIFILM Wako Pure Chemical Corporation, Osaka, Japan) was used. Similarly, 1000 μg mL^−1^ Pb, Rb, Sr, and Bi standard solutions (FUJIFILM Wako Pure Chemical Corporation, Osaka, Japan) were used to obtain the fitting parameters for the peaks corresponding to these elements.

### 2.2. Sampling of Seawater, Brackish Water, and River Water

**Figure 1:**
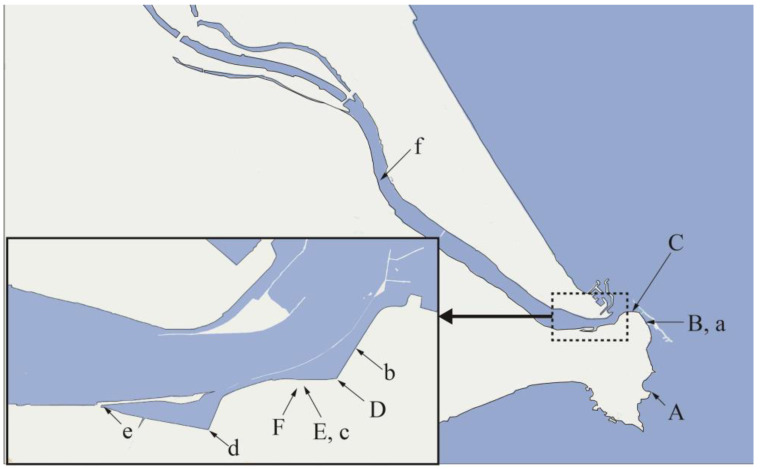
Sampling points. Sampling at the points shown in uppercase and lowercase letters was performed on different days. Points “B” and “a” and points “E” and “c” represent the same sampling points.

We selected the Tone River as a river unlikely to have been affected by the 2011 accident at the Fukushima Daiichi Nuclear Power Station caused by the Great East Japan Earthquake. The Tone River is the second longest river in Japan, and its estuary follows the border between the Chiba and Ibaraki prefectures. Samples of seawater, brackish water, and river water were collected twice along the coast of the Choshi Peninsula on the Chiba prefecture side. Figure 1 shows the sampling points, denoted by uppercase and lowercase letters to indicate sampling on different days.

Table 1 lists the salinity meter readings for the seawater, brackish water, and river water samples collected at each point. As there is no inflow of other rivers into the Tone River in this region, the salinity of the brackish water essentially correlates with the distance from the estuary, although some variations can be observed due to tidal factors. More specifically, water sampling at points “A” to “F” was performed at high tide, resulting in samples with high overall salinity. By contrast, water sampling at points “a” to “f” was performed at low tide, and the salinity levels of the samples were low. Points “B” and “a” and points “E” and “c” represent the same points, indicating sample collection at high and low tide, respectively. Moreover, points “A” and “f” correlate with the seawater and river water samples, respectively. Thus, six water samples (~250 mL each) were collected at each point and filtered through a 0.45 μm pore membrane filter. Additionally, 8 L of seawater was collected at point “A” and filtered as described above.

### 2.3. Sample Preparation

#### 2.3.1. Standard Samples for Examination of the Solid–Liquid Ratio

**Figure 2:**
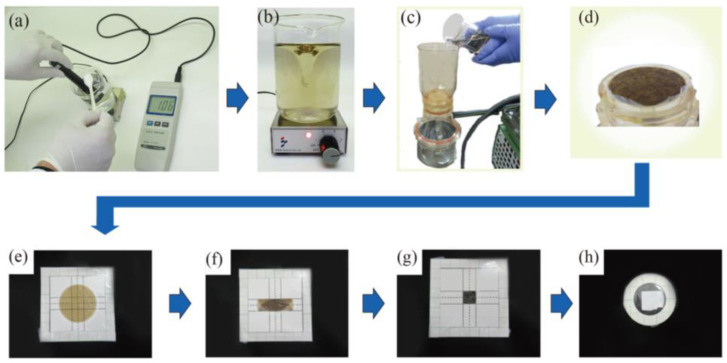
Outline of the sample preparation process. (**a**) Addition of sodium perchlorate monohydrate until the salinity meter reading reached 3%, followed by addition of the GO dispersion, and adjustment of the pH to 6. (**b**) Stirring of the sample solution. (**c**) Collection of GO using a membrane filter. (**d**) The membrane filter bearing the collected GO. (**e**) Placement of the GO collection membrane filter onto the lined mount. (**f**) Folding of the filter into 1/3 of its size in the vertical direction. (**g**) Folding of the filter into 1/3 of its size in the horizontal direction. (**h**) Sealing using a circular sealing material.

The preparation method employed herein was based on our previously reported study [17], and is summarized in Figure 2. Initially, sodium perchlorate monohydrate was added to 75, 150, 225, and 300 mL of pure water until the salinity meter reading reached 3% (*n* = 6). The lowest sample volume of 75 mL corresponded to that employed in our previous study [17]. Larger sample volumes (i.e., 2, 3, and 4 times larger) were also tested to examine the effect of the sample volume. Subsequently, the addition of XSTC-1407 (30 μL) to each solution afforded uranium concentrations of 4.0, 2.0, 1.3, and 1.0 ng mL^−1^, respectively. As the same amount of uranium was added to samples of different volumes, the signal intensities of the U Lα peak were expected to be equal if all of the uranium was collected by GO. Thus, the GO dispersion (600 μL) was added; the pH was adjusted to 6, as described in a previous study [19]; and each mixture was stirred for 10 min. The GO containing the adsorbed uranium was then collected by passing each of the above solutions through a PTFE membrane filter with a pore size of 0.1 μm. The resulting GO-containing membrane filter was placed on a mount with lines at a distance of 13 mm in the vertical and horizontal directions, then covered with a 0.3 µm thick Mylar film and folded along the lines to create a square sample with a side length of 13 mm. Subsequently, double-sided adhesive tape was attached to one side of a cardboard ring with an outer diameter of 40 mm and an inner diameter of 25 mm. Single-sided adhesive polypropylene tape (50 µm thickness) was attached on the other side to close the hole in the center. This ring was attached to the Mylar film to cover the folded GO-containing membrane filter, and the Mylar film was cut out along the edge of the ring to give a thin disk-shaped sample.

#### 2.3.2. River Water, Brackish Water, and Seawater Samples

An aliquot (200 mL) of each collected seawater, brackish water, and river water sample was dispensed into a beaker, and sodium perchlorate monohydrate was added until the salinity meter reading reached 3%. It should be noted here that sodium perchlorate was not added to the seawater collected at point “A” because the salinity value already exceeded 3% (viz. 3.2%). Following the addition of the GO dispersion (600 μL), the pH was adjusted to 6, as described in a previous study [19], and each mixture was stirred for 10 min. The GO containing the adsorbed uranium was then collected using the PTFE membrane filter, placed on the Mylar film, dried, folded, and sealed as described in Section 2.3.1.

#### 2.3.3. Standard Samples for the Standard Addition Method and the Calibration Curve Method

To confirm the uranium concentration in the seawater sample collected at point “A”, analysis was performed based on the standard addition method. More specifically, aliquots (200 mL) of the collected seawater were dispensed into 30 beakers, to which XSTC-1407 was added (0, 10, 20, 30, and 40 μL; *n* = 6) to give uranium masses of 0, 100, 200, 300, and 400 ng, respectively. All subsequent steps, from the addition of GO to the sealing of the membrane filter sample, were conducted as described above.

To obtain a calibration curve, standard samples were prepared. In this case, a sodium perchlorate aqueous solution (200 mL) with a salinity of 3% was dispensed into 36 beakers, to which XSTC-1407 was added (0, 10, 20, 40, 60, and 80 μL; *n* = 6) to give standard solutions with uranium concentrations of 0.0, 0.5, 1.0, 2.0, 3.0, and 4.0 ng mL^−1^, respectively. All subsequent steps, from the addition of GO to the sealing of the membrane filter sample, were conducted as described above.

#### 2.3.4. Standard Samples for Determination of the Fitting Parameters

To determine the fitting parameters, samples containing each individual element were prepared. According to our previous studies [17,22,23,24], a bromine solution (10 µL) with an adjusted concentration of 100 µg mL^−1^ was placed onto filter paper with a thickness of 220 µm (No. 5A, Toyo Roshi Kaisha, Ltd., Tokyo, Japan) that was cut to a diameter of 5.5 mm. After drying, the bromine-containing filter paper was covered with Mylar film and single-sided polypropylene adhesive tape. Similarly, samples containing only lead, bismuth, rubidium, and strontium were prepared. For uranium and thorium, the samples were prepared using XSTC-1407.

### 2.4. XRF Measurements

An Epsilon 4 tabletop XRF spectrometer (PANalytical, Malvern Instruments, Spectris Plc., London, UK), equipped with an Ag anode X-ray tube source and a silicon drift detector with an energy resolution of ~135 eV (Mn Kα), was used to perform the XRF measurements. As in our previous study, copper with a thickness of 300 µm was selected as the primary X-ray filter [17,25], and incident X-rays with diameter ranges of ~15 mm were used for irradiation. As the GO collection membrane filter was folded into a square with a side length of 13 mm, the majority of the membrane, with the exception of its four corners, was irradiated by the incident X-rays. A cylindrical stainless-steel weight was placed on the thin sample to prevent the sample from floating. The output power of the X-ray tube was 15 W, and the tube voltage and current were set to 50 kV and 0.3 mA, respectively. The X-ray accumulation time for each spectral measurement was 300 s. However, to obtain statistically relevant results and, therefore, to determine the optimal solid/liquid ratio and obtain the fitting parameters for the individual elements, an accumulation time of 3600 s was employed.

### 2.5. Peak Fitting

To obtain the peak intensities, multi-Gaussian fitting was performed as described in detail in our previous studies [17,22,23,24,25]. In brief, the key feature of this method is that a number of fitting parameters can be fixed in advance by means of actual measurements, in addition to using the peak central energy given in our previous study [26]. Peak fitting was performed only within the energy region of 12.2–14.5 keV, which corresponded to the Pb Lβ (12.61 keV), Bi Lβ (13.02 keV), Th Lα (12.97 keV), Br Kβ (13.29 keV), Rb Kα (13.40 keV), U Lα (13.61 keV), and Sr Kα (14.16 keV) peaks. Of these, the Pb Lβ peak is a combination of multiple peaks, including the Lβ_1_ (12.61 keV), Lβ_2_ (12.62 keV), Lβ_3_ (12.80 keV), and Lβ_4_ (12.31 keV) peaks. As the Lβ_1_ and Lβ_2_ peaks are extremely close to one another, they can be considered a single peak. Thus, the Pb Lβ peak was regarded as an overlap of the Lβ_1_ + Lβ_2_, Lβ_3_, and Lβ_4_ peaks. Similarly, the Bi Lβ peak was also considered to be an overlap of three peaks, namely Bi Lβ_1_ (13.02 keV) + Lβ_2_ (12.98 keV), Lβ_3_ (13.21 keV), and Lβ_4_ (12.69 keV). The U Lα peak consists of both the Lα_1_ (13.61 keV) and Lα_2_ (13.44 keV) peaks. Similarly, the Th Lα peak consisted of both the Lα_1_ (12.97 keV) and Lα_2_ (12.81 keV) peaks. Although the Rb Kα and Sr Kα peaks also had two components (i.e., Rb Kα_1_ (13.39 keV) and Rb Kα_2_ (13.36 keV) and Sr Kα_1_ (14.16 keV) and Sr Kα_2_ (14.10 keV)), the Rb Kα peak could be considered a single peak due to the minimal separation between the peaks. Because the fitting did not converge unless restrictions were applied to these peaks, some parameters were fixed as follows. Initially, the peak central energy was fixed to previously reported values [18], and the quadruple-Gaussian fitting for the XRF spectrum of a small piece of filter paper containing only XSTC-1407 was used to provide the widths of the U Lα_1_, U Lα_2_, Th Lα_1_, and Th Lα_2_ peaks, in addition to the intensity ratio of the U Lα_2_ peak compared to the U Lα_1_ peak, and that of the Th Lα_2_ peak compared to the Th Lα_1_ peak. Similarly, the widths of the Lβ_1_ + Lβ_2_, Lβ_3_, and Lβ_4_ peaks, and the intensity ratios of the Lβ_3_ and Lβ_4_ peaks compared to the main peak (i.e., the Lβ_1_ + Lβ_2_ peak), were fixed for lead and bismuth by triple-Gaussian fitting of the XRF spectra obtained from small pieces of filter paper containing lead or bismuth. Single Gaussian fitting for the XRF spectra of the small pieces of filter paper containing the rubidium standard or the bromine solution gave the widths of the Rb Kα and Br Kβ peaks, respectively. Double Gaussian fitting for the XRF spectra of the filter paper specimen containing the strontium standard gave the widths of the Sr Kα_1_ and Kα_2_ peaks, as well as the intensity ratio of the Sr Kα_2_ peak compared to that of the Sr Kα_1_ peak.

After fixing these parameters, the fitting parameters represented only the background and intensity of the main peak of each element. The net signal intensity of each peak can, therefore, be obtained from the area of the reproduced Gaussian function, and the net intensity of the U Lα peak can be considered as the sum of the net intensities of the U Lα_1_ and Lα_2_ peaks. The energy range of each peak was set to 3 times the full width at half maximum. The background intensity was obtained as the area of the background function in peak fitting within the same energy range, and not by measurement of the blank sample. The results of multiple samples are represented by their mean and standard deviation values.

## 3. Results

### 3.1. Measured XRF Spectra for the Standard Sample

**Figure 3:**
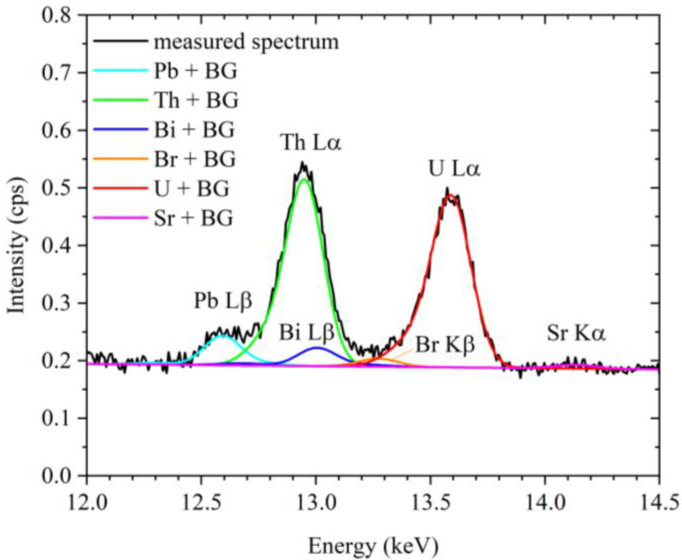
Measured XRF spectrum for one of the standard samples. BG = background intensity.

Figure 3 shows an enlarged view of the measured XRF spectrum for the standard sample prepared using the 4 ng mL^−1^ uranium solution (75 mL). In the observed energy region, the Pb Lβ, Bi Lβ, Th Lα, Br Kβ, Rb Kα, U Lα, and Sr Kα peaks can be observed; notably, the Pb Lβ, Th Lα, and U Lα peaks are the most intense, and an extremely weak peak corresponding to Sr Kα is observed. It should be noted that the Pb Lβ and Sr Kα peaks were ascribed to lead and strontium impurities in the GO synthesized from natural graphite, as described in our previous study [17].

The result of fitting according to the method described in Section 2.5 is also shown in Figure 3. In addition to the components of the Pb Lβ, Th Lα, U Lα, and Sr Kα peaks, those of the Bi Lβ and Br Kβ peaks were also revealed. As described in our previous study [17], the copper primary X-ray filter contained a small amount of bismuth, thereby accounting for the presence of the Bi Lβ peak. In addition, as the current study was conducted in a general laboratory rather than in a clean room, it was difficult to prevent contamination from naturally occurring bromine, resulting in the overlapping of the Br Kβ peak with the U Lα peak. As a result of peak fitting, the intensity of the Rb Kα peak was zero.

**Figure 4:**
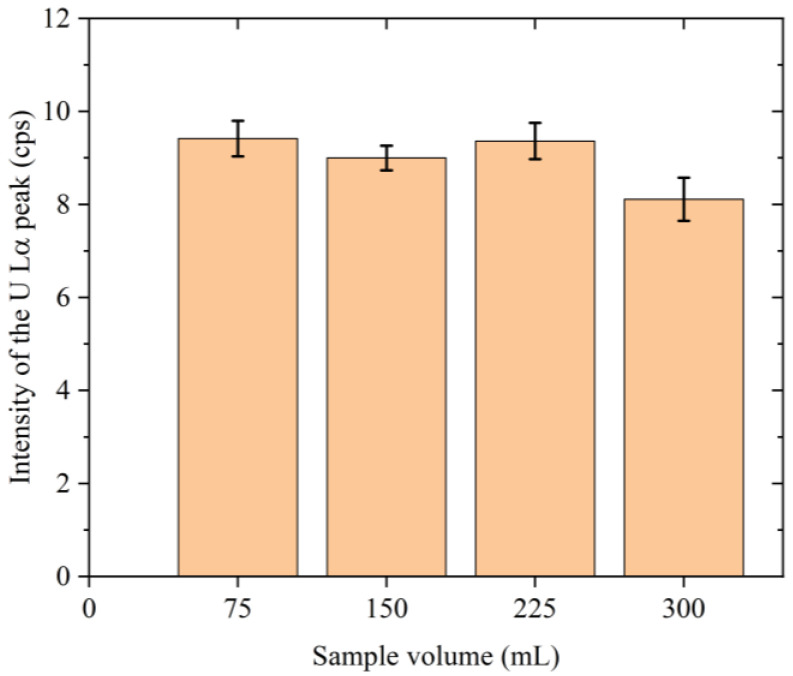
Relationship between the sample volume and the intensity of the U Lα peak for the standard sample. The error bars represent the standard deviations of the results obtained from six samples.

Figure 4 shows the relationship between the volume of the standard sample and the net intensity of the U Lα peak obtained from the area of the reproduced Gaussian function. The error bars in this figure represent the standard deviations of the results obtained from six samples. As indicated, for the samples based on standard solution volumes of 75, 150, and 225 mL, the net intensities of the U Lα peak were within the error margins. Although the intensity of the corresponding peak in the 300 mL solution was significantly lower, the sample volume of 200 mL used in this study is within the range in which no change in the intensity of the U Lα peak was observed.

### 3.2. XRF Spectra of the River Water, Brackish Water, and Seawater Samples

**Figure 5:**
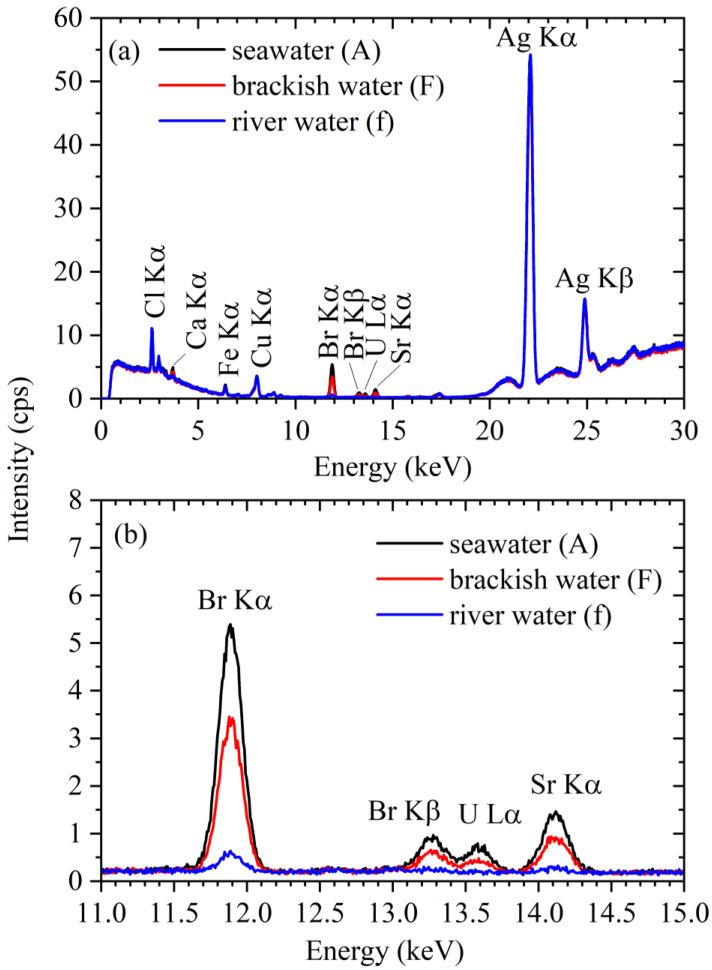
XRS spectra of the samples of river water (collected at point “A”), brackish water (collected at point “F”), and seawater (collected at point “f”). (**a**) The whole spectra; (**b**) an enlarged view.

Figure 5a shows the XRF spectra recorded for the seawater, brackish water, and river water samples collected at points “A”, “F”, and “f”, respectively. In these three spectra, several general features were found to be similar, including the background signal; the Ag Kα (22.16 keV) and Kβ (24.94 keV) peaks, derived from the target of the X-ray tube; the Cu Kα (8.05 keV) peak, derived from the primary X-ray filter; and the Cl Kα (2.62 keV) peak, derived from the sea salt and the added sodium perchlorate monohydrate. By contrast, the intensities of the Ca Kα (3.69 keV), Br Kα (11.92 keV), Br Kβ, U Lα, and Sr Kα peaks appeared to correlate with the solution salinity. Figure 5b shows an enlarged view of Figure 5a. From this enlargement, the Br Kα, Br Kβ, U Lα, and Sr Kα peaks can be clearly observed in the spectra of the seawater and brackish water samples, while in the spectrum recorded for the river water sample, only the Br Kα peak can be clearly observed.

### 3.3. Relationship between the Salinity and the Signal Intensity

**Figure 6:**
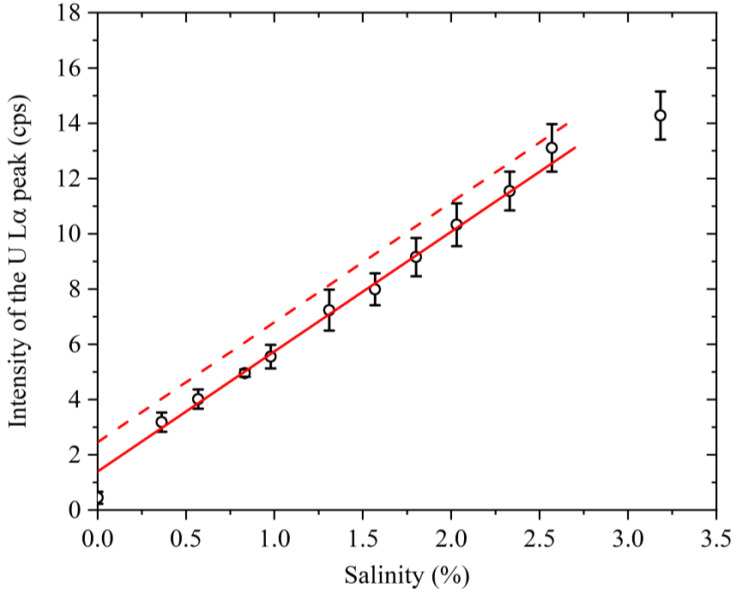
Relationship between the salinity and intensity of the U Lα peak. The error bars represent the standard deviations of the results obtained from six samples. The solid line is the result of linear fitting of the data set with a salinity range of 0.57–2.57%. The dotted line is the result of a vertical shift by an amount of signal intensity (1.1 cps) corresponding to the detection limit (DL).

The net intensities of the U Lα peaks obtained using the peak fitting method are listed in Table 1, and the resulting relationship between the solution salinity and the net U Lα peak intensity is presented in Figure 6. With the exception of the data correlating to the lowest salinity (i.e., river water) and the highest salinity (i.e., seawater), linear relationships were observed. More specifically, the solid red line in Figure 6 shows the result of linear fitting (*R*^2^ = 0.986) for the brackish water data (i.e., excluding the river water and seawater samples collected at points “A” and “f”).

### 3.4. Determination of the Uranium Concentration in Seawater

**Figure 7:**
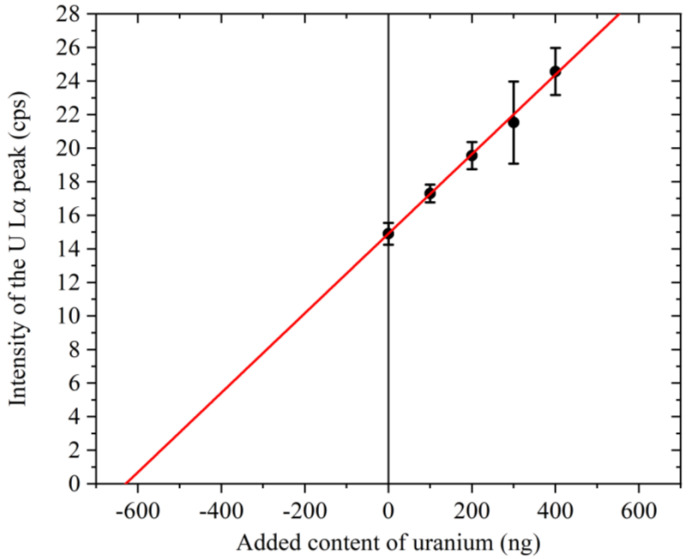
Result of the standard addition method for seawater. The *x*-coordinate of the point where the straight line intersects the horizontal axis is approximately −630 ng. The error bars represent the standard deviations of the results obtained from six samples.

As shown in Figure 7, using the standard addition method, the uranium content determined by extrapolation was ~630 ng, thereby giving a uranium concentration of ~3.15 ng mL^−1^ in a 200 mL volume of seawater. The error bars in the figure represent the standard deviations of the results obtained from six samples, and it is probably coincidental that the error bars for the uranium additions of 300 and 400 ng are larger than those for the other data points. This result is in good agreement with previously reported values [9].

### 3.5. Calibration Curve and Determination of the DL

A calibration curve was constructed using the spectra recorded for the standard samples. It was revealed that the net intensity of the U Lα peak obtained by peak fitting was directly proportional to the uranium concentration (data not shown). The slope of the calibration curve was 6.33 cps ng^−1^ mL (*R*^2^ = 0.999).

As described in our previous studies [17,22,23,24], the DL of uranium can be calculated using Equation (1):

(1) $$\mathrm{DL}=\frac{3c}{I_{\mathrm{net}}}\sqrt{\frac{I_{\mathrm{BG}}}{t}},$$

where *I*_net_ is the net intensity of the U Lα peak (cps), *I*_BG_ is the background intensity in the same energy region (cps), *c* is the concentration of uranium (ng mL^−1^), and *t* is the measurement time (s). The DL is usually obtained from the results recorded for the sample with the lowest concentration of uranium (i.e., 0.5 ng mL^−1^). Thus, because six samples containing this concentration of uranium were available, six values could be determined for the DL. Using the obtained values, the maximum DL was taken, giving a value of 0.17 ng mL^−1^ when considering the variability of the parameters determined via peak fitting. The intensity of the U Lα peak corresponding to the DL was measured as 1.1 cps.

Using the calibration curve, the intensity of the U Lα peak for the 200 mL solution with a uranium concentration of 3.15 ng mL^−1^ was determined to be 19.9 cps, which is the expected value for a pure uranium solution without matrix components.

## 4. Discussion

During this study, we initially examined the degree by which the sample volume could be increased under the same experimental conditions as those described in our previous study. Based on the obtained results, seawater, brackish water, and river water samples were analyzed, and the relationship between the salinity and U Lα peak signal intensity was obtained. Using this relationship, we proposed a method for facile screening to determine the presence or absence of uranium contamination in brackish water following an accident, and the applicable range of this method was evaluated.

As shown in Figure 4, the net intensities of the U Lα peaks for the samples prepared using 75, 150, and 225 mL of sodium perchlorate and equal amounts of uranium were within the error margin. Our previous study examined the amount of GO added to a solution containing 300 ng uranium (75 mL) and the efficiency of uranium collection, and the results showed that 2.4 mg of GO was sufficient for complete uranium adsorption [22]. These results demonstrate that in a sample with a volume of ≤225 mL, a small quantity of GO present in the dispersion (2.4 mg in 600 μL) was sufficient to adsorb the uranium present in the sample. For ease of measurement, we therefore selected a sample volume of 200 mL for analysis of the river water, brackish water, and seawater specimens. It should be noted that this result may differ when using different GO than that of the commercial GO nanosheets we used.

In terms of the relationship between the salinity and uranium concentration, it was revealed that the intensity of the U Lα peak exhibited a linear relationship with the salinity over a salinity range of 0.57–2.57% (Figure 6). Thus, within the linear range, measurement of the salinity of the brackish water sample would allow for the estimation of the intensity of the U Lα peak derived from the originally present uranium. After the accident, the brackish waters should be sampled at several points and the salinity should be measured to predict the net intensity of the U Lα peak derived from the originally present uranium. When the obtained net intensity of the U Lα peak shows an increase beyond 1.1 cps, as indicated by the vertical shift of the solid line in Figure 6, uranium contamination can be expected after an accident. More specifically, if the plot obtained for the sampled brackish water is located above the dotted line (Figure 6), uranium contamination is considered to be present. It should be noted that the DL of the current method is 0.17 ng mL^−1^, which is 4.3 μBq cm^−3^ when converted to a radioactivity concentration based on the isotopic composition ratio of natural uranium. In Japan, the regulatory limit for the major uranium isotopes (i.e., ^234^U, ^235^U, and ^238^U) in effluents is 20 mBq cm^−3^ [27]; importantly, the DL of our method is four orders of magnitude lower than this regulatory value. Thus, the current method can detect the presence of uranium in brackish water at levels slightly higher than the naturally expected content.

As shown in Figure 6, the plot corresponding to the river water sample collected at point “f” does not fall on the solid straight line, and this was attributed to a salinity reading of 0%. More specifically, upon tracing the river from the estuary to the upstream area, both the salinity and the signal intensity of the U Lα peak decreased at constant rates. When the salinity reached 0%, the signal intensity of the U Lα peak matched the vertical intercept of the solid straight line. Further upstream, the salinity remained at 0% and the signal intensity of the U Lα peak further decreased. In other words, using this method, it was not possible to determine the amount of uranium originally present in river water with a salinity reading of 0%, and only the upper limit could be obtained. However, as the subject of this research is brackish water, this was not considered a significant issue.

In addition, the plot corresponding to the seawater sample collected at point “A” fell below the solid straight line. Upon analysis of this sample using the standard addition method (Figure 7), the estimated net intensity of the U Lα peak for a pure solution with a uranium concentration of 3.15 ng mL^−1^ and without matrix components was determined to be 19.9 cps. However, as shown in Table 1, the net intensity of the U Lα peak for seawater sampled at point “A” was 14.3 cps. This lower value was considered to originate from the inhibition of uranium adsorption onto GO due to the presence of various interfering matrix components in the sample solution.

Furthermore, Figure 6 shows that the expected signal intensity for the U Lα peak upon extrapolation of the solid straight line to a salinity of 3.15% was lower than the expected value for a pure uranium solution (19.9 cps). This indicates that a reduction in the signal intensity due to the inhibition of uranium adsorption onto GO also occurred in brackish water due to the presence of matrix components, as mentioned above. In the salinity region of 0.57–2.57%, the salinity and the signal intensity of the U Lα peak were proportional to one another. It was, therefore, assumed that the magnitude of the inhibition of uranium adsorption was also proportional to the salinity. This is consistent with the intuitive prediction that many of the matrix components derive from seawater, meaning that their concentrations will be proportional to the seawater content, as in the case of salinity. However, it was also revealed that the inhibition of uranium adsorption was even greater close to the estuary, where the salinity was between 2.57–3.2%, and this was attributed to the complex behavior of seawater and river water at this point [15]. As shown in Figure 1, point “B”, where brackish water with a salinity of 2.57% was collected, is adjacent to the estuary, meaning that the area beyond this point can be considered as seawater. In seawater areas, even when uranium derived from accidents is incorporated, it is rapidly diffused by the tidal currents, making the detection of uranium contamination using this method impossible. In other words, such areas are also outside the scope of this study.

Based on the above results, it is apparent that our method is not applicable for river water and seawater close to the estuary; however, in the case of brackish water with a salinity of ~0.5–2.5%, the salinity levels can be used to estimate the XRF signal intensity for the originally present background uranium and to determine if uranium contamination due to an accident is present. A key feature of this method is that the presence or absence of contamination can be determined without measuring the uranium concentration in brackish water. This method should also be applicable to the analysis of samples from any river, as the matrix component concentrations in seawater are considered to be similar worldwide. In other words, the slope of the straight line shown in Figure 6 is expected to be comparable for all rivers. Moreover, this method can be used to estimate the XRF signal intensity of uranium derived from seawater, not only in brackish water, but also in liquid samples containing seawater. As the contaminated water at the Fukushima Daiichi Nuclear Power Station includes seawater, this approach may serve as a simple method for determining whether the contamination levels have risen, with respect to the original values, due to decommissioning work.

## 5. Conclusions

In this study, an XRF method was developed for the facile screening of uranium in brackish water. This method was proven to be suitable for determining increases in uranium concentration attributed to a nuclear accident. Because the signal intensity of the U Lα peak originating from the background uranium originally present in the brackish water can be estimated based on its proportional relationship to the sample’s salinity, a significant increase in the intensity of the U Lα peak can be attributed to the presence of uranium derived from accidents. Even though the precise analysis of these samples is desirable, more sensitive analytical methods, such as inductively coupled plasma mass spectrometry, are significantly affected by high salinity in samples. Our proposed XRF technique is not affected by salinity, thereby indicating its suitability for the facile screening of uranium contamination. Moreover, this method may be applicable to other samples containing seawater. Thus, future work should include an evaluation of the applicability of the presented method to the decommissioning work at the Fukushima Daiichi Nuclear Power Station.

## Figures and Tables

**Table 1:** Salinity values and U Lα peak signal intensities at the various sampling points.

Sampling Point	Salinity (%)	U Lα (cps)
A	3.18	14.3 ± 0.9
B	2.57	13.1 ± 0.9
C	2.33	11.5 ± 0.7
D	2.03	10.3 ± 0.8
E	1.80	9.2 ± 0.7
F	1.57	8.0 ± 0.6
a	1.31	7.2 ± 0.7
b	0.98	5.6 ± 0.4
c	0.83	5.0 ± 0.1
d	0.36	3.2 ± 0.3
e	0.57	4.0 ± 0.4
f	0.00	0.4 ± 0.2

## Data Availability

The data presented in this study are available upon request from the corresponding author.
